# Supplementary material for: Origin of interfacial perpendicular magnetic anisotropy in MgO/CoFe/metallic capping layer structures
Source: Sci Rep. 2015 Dec 11;5:18173. doi: 10.1038/srep18173 (PMC4676065; doi:10.1038/srep18173)
Supplement: Supplementary Information [file srep18173-s1.pdf]

*Supplementary Information*

# Origin of interfacial perpendicular magnetic anisotropy in MgO/CoFe/metallic capping layer structures

**Shouzhong Peng[1,2], Mengxing Wang[1,2], Hongxin Yang[3], Lang Zeng[1,2], Jiang Nan[1,2], Jiaqi Zhou[1,2], Youguang Zhang[1,2], Ali Hallal[3], Mairbek Chshiev[3], Kang L. Wang[4], Qianfan Zhang[1,5,#], Weisheng Zhao[1,2,*]**

[1] Fert Beijing Institute, Beihang University, Beijing 100191, China

[2] School of Electronic and Information Engineering, Beihang University, Beijing 100191, China

[3] Univ. Grenoble Alpes, INAC-SPINTEC, F-38000 Grenoble, France; CEA, INAC-SPINTEC, F-38000 Grenoble, France and CNRS, SPINTEC, F-38000 Grenoble, France

[4] Department of Electrical Engineering, University of California, Los Angeles, California 90095, USA

[5] School of Materials Science and Engineering, Beihang University, Beijing 100191, China

[*]E-mail: weisheng.zhao@buaa.edu.cn

[#]E-mail: qianfan@buaa.edu.cn

## Interaction between the MgO/CoFe and CoFe/Ta interfaces with different CoFe thicknesses

In order to investigate the coupling between the MgO/CoFe and CoFe/capping layer interfaces, we calculate the interfacial magnetic anisotropy constant $K_i$ of the MgO/CoFe/Ta structure with the CoFe thickness being three, five and nine monolayers. The results are shown in Table S1. It can be found that there is only a slight decrease in the magnetic anisotropy energy (MAE) value when the CoFe thickness reduces from nine monolayers to five monolayers. However, when the CoFe layer is three

monolayers, the MAE value diminishes to 1.21 erg/cm$^2$, which indicates a much stronger interference effect between the MgO/CoFe and the CoFe/Ta interfaces. In order to further verify this interaction, we show the projected density of states (PDOS) in Fig. S1. As we can see in Fig. S1(a) and (b), in the vicinity of the Fermi energy ($E_F$), PDOSs of Co atoms in the MgO/CoFe/Ta system are much different from that in the MgO/CoFe system. For example, a great change can be observed in the PDOS of $d_{xy}$ and $d_{yz}(d_{xz})$ orbitals in the energy range of -2 to 2 eV. These changes clearly prove that the Ta capping layer has an effect on the MgO/CoFe interface when the CoFe thickness is three monolayers. A similar conclusion that the MgO layer has influence on the CoFe/Ta interface can be drawn by comparing Fig. S1(c) and (d). In conclusion, there is an obvious coupling between the MgO/CoFe and CoFe/Ta interfaces in the MgO/CoFe/Ta structure with three CoFe monolayers, which leads to a decrease in perpendicular magnetic anisotropy (PMA) value, while there is almost no interaction between them when the CoFe thickness becomes nine monolayers.

| CoFe thickness | 3 monolayers | 5 monolayers | 9 monolayers |
|---|---|---|---|
| $K_i$ (erg/cm$^2$) | 1.21 | 1.70 | 1.77 |

Table S1. Calculated MAE values of the MgO/CoFe/Ta structure with the CoFe thickness being three, five and nine monolayers.

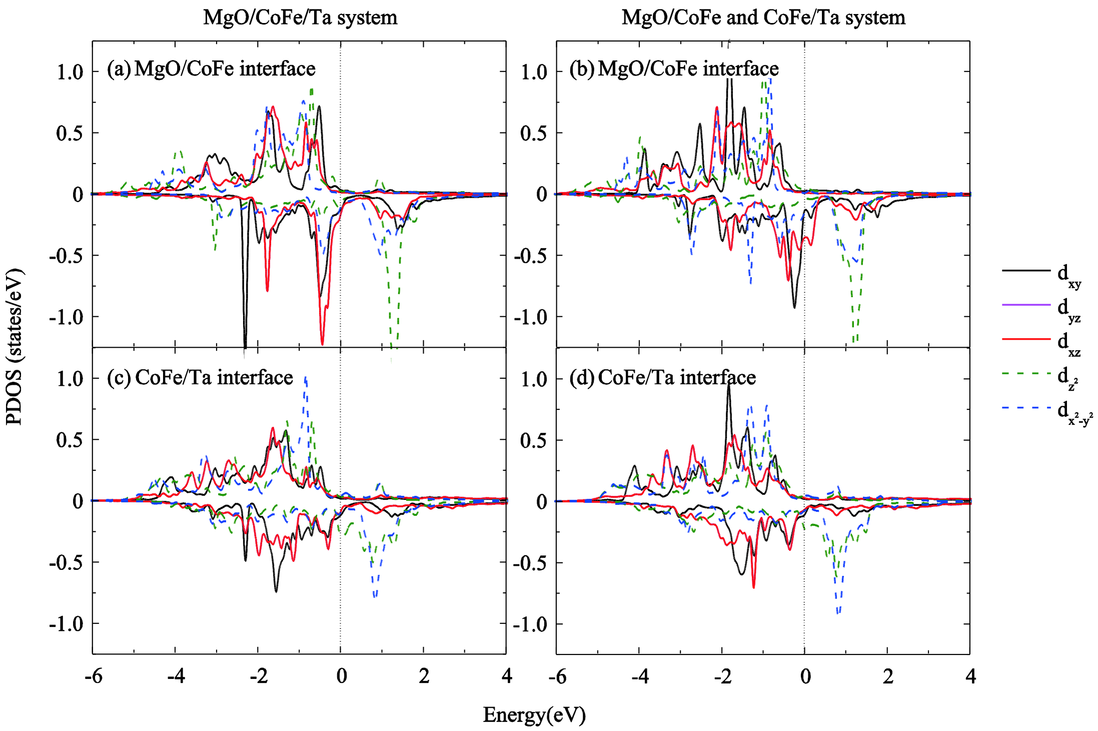


Figure S1. Majority-spin (positive) and minority-spin (negative) PDOS on the *d* orbitals of Co atom in the MgO/CoFe interface of (a) MgO/CoFe/Ta system and (b) MgO/CoFe system, and in the CoFe/Ta interface of (c) MgO/CoFe/Ta system and (d) CoFe/Ta system with the CoFe thickness being three monolayers. The zero of energy

is set to be $E_F$.

## Analysis of the CoFe/X interfacial configuration

Six configurations of the CoFe/X (X=Ru, Ta and Hf) interface are considered as shown in Fig. S2 (b-d). The Co-terminated hollow structure is found to be the most energetically preferable structure. In Table S2 we present the total energy of the relaxed CoFe/Ta structures with different interfacial configurations. For both Co-terminated and Fe-terminated structures, the hollow site is the most energetically favorable by 0.74-1.24 eV/cell. Moreover, in order to compare the interfacial stability of the Co-terminated and Fe-terminated structures, we calculate the binding energy $E_b$, which is given by:

$$E_b = E(\text{CoFe slab}) + E(\text{Ta slab}) - E(\text{CoFe/Ta structure}) \quad \text{(S1)}$$

where 'slab' refers to thin film extracted from CoFe/Ta structures with no further atomic relaxation (i.e., atoms in slabs are kept at the equilibrium positions in the relaxed CoFe/Ta structure for the slab calculations). As shown in Table S3, the Co-terminated hollow site has a largest binding energy of 2.61 eV, which implies that this is the optimal configuration because of the strongest binding at the CoFe/Ta interface. The same conclusion was drawn for CoFe/X (X=Ru and Hf) structures after the calculations of these two systems.

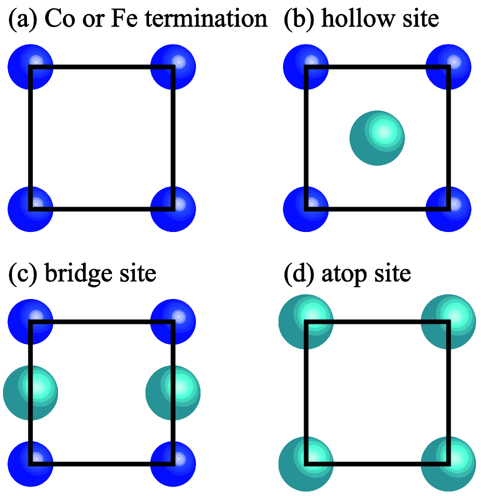


Figure S2. Top view of (a) (1×1) unit cell of CoFe(001) surface with Co or Fe termination, and CoFe/X(001) interface for (b) hollow, (c) bridge and (d) atop adsorption sites of X atoms, with CoFe[100] parallel to X[110] direction. The blue balls represent Co or Fe atoms, and the green balls represent X atoms. In the atop site structure, Co or Fe atoms are covered by the X atoms and cannot be seen.

| | hollow | bridge | atop |
|---|---|---|---|
| Co-terminated | -124.70 | -123.96 | -123.46 |
| Fe-terminated | -125.60 | -124.85 | -124.40 |

Table S2. Total energy (in eV) of CoFe/Ta structures with different interfacial configurations.

| | hollow | bridge | atop |
|---|---|---|---|
| Co-terminated | 2.61 | 1.85 | 1.34 |
| Fe-terminated | 2.42 | 1.68 | 1.22 |

Table S3. Binding energy (in eV) of CoFe/Ta structures with different interfacial configurations.
